# Interventions to improve adherence to antenatal and postnatal care regimens among pregnant women in sub-Saharan Africa: a systematic review

**DOI:** 10.1186/s12884-020-02992-y

**Published:** 2020-05-24

**Authors:** Kristina Esopo, Lilly Derby, Johannes Haushofer

**Affiliations:** 1 Department of Counseling, Clinical, and School Psychology, University of California, Santa Barbara, Santa Barbara, 93106 CA USA; 2 Center for Behavioral Cardiovascular Health, Columbia University Medical Center, 630 West 168th Street, New York, 10032 NY USA; 3 Department of Psychology, Princeton University, 427 Peretsman-Scully Hall, Princeton, 08544 NJ USA; 4 Woodrow Wilson School for Public and International Affairs & Department of Economics, Princeton University, Princeton, 08544 NJ USA

**Keywords:** Antenatal care, Postnatal care, Adherence, Sub-Saharan Africa, Interventions, Systematic review

## Abstract

**Background:**

Pregnant women in sub-Saharan Africa tend to have low adherence to antenatal and postnatal care regimens, contributing to high infant and child mortality rates. Despite low adherence figures and the high returns from attending antenatal and postnatal care visits, research on interventions to improve adherence is in its infancy. Our aim was to determine the effectiveness of existing interventions to improve adherence to antenatal and postnatal care regimens among pregnant women in sub-Saharan Africa.

**Methods:**

Full text, peer-reviewed articles, published in English and listed in PubMed or PsycINFO through January 2018 were identified in a systematic review. Studies were restricted to randomized controlled trials only and had to assess intervention impact on antenatal and postnatal care adherence, operationalized as the frequency of visits attended. Two reviewers independently screened papers for inclusion and evaluated the risk of systematic error in each study using the Cochrane risk of bias tool. Any discrepancies were reconciled by a third independent reviewer.

**Results:**

The initial search generated 186 articles, of which, five met our inclusion criteria. Due to the small sample size and methodological variation across studies, a pooled effect size estimate could not be obtained. Therefore, effects on antenatal and postnatal care adherence were examined and reported at the individual study level. None of the interventions were directly aimed at improving adherence, but two of the five, both behavioral interventions, demonstrated effectiveness in increasing antenatal care (rate ratio 5.86, 95% CI 2.6-13.0, p<0.0001) and postnatal care adherence (31.3%, 95% CI 15.4-47.2, p=0.0009), respectively. Three home visit interventions had no effect on antenatal care adherence. Although the risk of bias was unclear or high in some cases, it remained low in most categories across studies.

**Conclusions:**

Results point to a large gap in the literature on interventions to address antenatal and postnatal care adherence in sub-Saharan Africa. Interventions drawing upon the executive function literature and the promising results of the behavioral interventions reviewed here are urgently needed to address these gaps.

**Trial registration:**

The review was prospectively registered with PROSPERO, id number https://www.crd.york.ac.uk/prospero/display_record.php?RecordID=88152, on February 7, 2018.

## Background

The infant mortality rate, defined as the probability of dying before age 1, was 31 deaths per 1,000 live births worldwide in 2016, with 61% occurring in the first 28 days of life [1]. Despite improvement in the last two decades, the global maternal mortality rate remains at 216 deaths per 100,000 live births [2]. These figures are highest in sub-Saharan Africa, where the infant mortality rate is 53 deaths per 1,000 live births; the neonatal mortality rate is 28 deaths per 1,000 live births; and the maternal mortality rate is 547 deaths per 100,000 live births [1].

One factor hypothesized to play an important role in accounting for regional disparities in infant and maternal mortality is antenatal (ANC) and postnatal care (PNC) regimen takeup, as high quality ANC and PNC are thought to significantly improve maternal and newborn health outcomes. For instance, regular contact with a skilled doctor, nurse, or midwife during ANC allows pregnant women to prepare for delivery and receive education on the warning signs of poor maternal or infant health during pregancy and childbirth. In addition, assessment of the mother and baby during PNC is vital to mitigate the risks to maternal and infant morbidity and mortality, which are highest in the days and weeks following childbirth. In a systematic review, Darmstadt et al. [3] review the evidence for the effect of 16 ANC, intrapartum, and PNC interventions on neonatal survival and suggest that universal coverage of these interventions has the potential to prevent an estimated 41-72% of neonatal deaths worldwide. While there is a dearth of causal evidence on the effectiveness of these interventions in sub-Saharan Africa, a recent meta-analysis demonstrated that ANC attendance was associated with lower neonatal mortality across most regions in low- and middle-income countries, including sub-Saharan Africa [4]. Further, another study in Kenya found that the highest rates of neonatal mortality were among neonates whose mothers did not attend any ANC visit and lacked skilled ANC attendance during pregnancy [5]; approximately 38% of all neonatal deaths in Kenya were deemed to be attributable to lack of check-ups for pregnancy complications, suggesting potential high returns from attending high quality ANC in sub-Saharan Africa.

The World Health Organization (WHO) recommends that pregnant women attend a minimum of eight ANC visits with a skilled health provider and seek PNC check-ups within 24 hours, and no later than two days, after delivery [6, 7]. Until recently, the recommendation was 4 (not 8) visits; we focus on this recommendation because it was current when the studies we review were conducted. While more than 90% of women in developed regions, such as the Americas and Europe, adhere to the WHO’s previous ANC/PNC recommendations, only 52% in sub-Saharan Africa receive at least four ANC visits [8], and only 41% attend a PNC check-up within two days of childbirth [9]. Thus, low adherence to recommended ANC/PNC regimens in sub-Saharan Africa poses a significant risk to infant and maternal mortality.

In light of these low adherence figures and the high returns from attending ANC/PNC visits, it is perhaps surprising that research on interventions to improve adherence is in its infancy. In particular, because mothers are likely to be highly motivated to optimize their children’s outcomes, and ANC resources are often available even in resource-poor settings, it is possible that behavioral factors are obstacles to adherence. Our aim in the current review is to summarize all randomized controlled trials (RCTs) of behavioral interventions to increase adherence to ANC and PNC regimens among pregnant women in sub-Saharan Africa. Adhering to the Preferred Reporting Items for Systematic reviews and Meta-Analyses (PRISMA) guidelines [10] (see Additional file 1 for complete PRISMA checklist), we present and assess the results of each individual intervention study within a framework of psychological mechanisms hypothesized to affect adherence. We also examine the risk of bias at the study design level by rating the quality of the intervention studies reviewed. We conclude by making recommendations on how to use this review to inform the development and evaluation of future ANC/PNC adherence interventions.

## Methods

### Search strategy and selection criteria

Our aim was to determine the effectiveness of existing interventions to improve adherence to ANC and PNC regimens among pregnant women in sub-Saharan Africa. In January 2018, a systematic review protocol was registered with the international prospective registrar of systematic reviews, PROSPERO, with registration id number CRD42018088152 [11] (see Additional file 2 for published protocol). We identified published studies in the electronic databases of PubMed and PsycINFO. RCTs of interventions intended to improve adherence to the recommended number of ANC and/or PNC visits were sought and selected if they included pregnant women in sub-Saharan Africa and reported treatment effects on the primary outcome of interest, frequency of ANC/PNC visits attended. Publications were included regardless of whether ANC/PNC adherence was measured as a primary or secondary outcome of interest. We did not restrict publication date, but did limit the search to English-language and peer-reviewed articles. The search was further restricted to RCTs conducted only in sub-Saharan Africa to evaluate the causal effect of each intervention on ANC/PNC regimen adherence specific to this population. No further exclusion criteria were applied.

Three blocks of index terms were used to search the PubMed database, and four blocks to search PsycINFO for relevant articles through January 2018. The first block referred to *interventions* with terms including: “Intervention”, “Program”, and “Training”. To generate a comprehensive list of interventions targeting pregnant women in sub-Saharan Africa, the second block individually listed the names of every country in *sub-Saharan Africa*, i.e. “Botswana”, “Ethiopia”, “Kenya”, “Nigeria”, etc. The third block referred to *ANC/PNC adherence* with terms including: “Prenatal”, “Postnatal”, “Antenatal”, “Pregnant”, and “Adherence”. Due to an inability to restrict the sample to RCTs only in PsychINFO, a fourth block was added to the PsycINFO search strategy related to *RCT design* and included the following terms: “Randomized controlled trial”, “Randomized trial”, “RCT”, and “Randomized”.

For the initial search, two reviewers (KE, LD) used a software called abstrackr [12] to independently screen abstracts and subsequently accept or reject each study for full text review. Following our Population Intervention Comparison Outcome (PICO) search strategy [13], abstracts were rejected if the studies did not have (1) interventions that were geared toward pregnant women in sub-Saharan Africa, (2) at least one quantitative ANC/PNC adherence outcome measure, and (3) an RCT study design. Any disagreements regarding the eligibility of particular studies were resolved through discussion with a third independent reviewer (JH).

### Data analysis

The same two reviewers (KE, LD) independently reviewed the full text of the studies identified in the abstract screening phase and used a standardized, pre-piloted digital spreadsheet to extract data from all included studies. The following data were extracted: publication title and authors; study setting; study population and characteristics at baseline; study design; recruitment procedures; study completion rates; details of the intervention and control conditions, including number of participants assigned to each group; description of outcomes measured and times of measurement; and treatment effects. The extracted data were then used to determine study eligibility for inclusion in the review. Discrepancies between the data extracted and the final determination to include or exclude a particular study were reconciled by the third independent reviewer (JH).

In addition, each included study was assessed for risk of bias at the study design level that could potentially lead to underestimation or overestimation of the true treatment effect [14]. Using the Cochrane risk of bias tool [15], the same two reviewers (KE, LD) identified and recorded any information that was given about: (1) the randomization sequence generation (selection bias); (2) concealment of the treatment allocation sequence (selection bias); (3) blinding of participants and study personnel to treatment allocation (performance bias); (4) blinding of enumerators assessing outcomes and analyzing data to treatment allocation (detection bias); (5) participant exclusions, attrition, and incomplete outcome data (attrition bias); (6) selective outcome reporting (reporting bias); and (7) other sources of bias, such as baseline imbalance, recruitment issues, etc. Qualitative ratings of “low risk”, “high risk”, or “unclear risk” were given for each of these internal validity indicators within studies. Criteria for each rating were determined according to the *Cochrane Handbook for Systematic Reviews of Interventions* [16].

For random sequence generation, studies were considered to have a “low risk” of bias if the sequence was generated using a random number table, computer random number generator, stratified or block randomization, minimization, or a low tech method (e.g. coin toss, shuffling cards or envelopes, throwing dice). Studies were considered to have a “high risk” of bias if the sequence was generated using quasi-random (e.g. date of birth, day of visit, ID number) or non-random methods (e.g. choice of clinician or participant, test results, availability).

For allocation concealment, studies were considered to have a “low risk” of bias if the treatment allocation occurred using central randomization (i.e. site was remote from trials location); sequentially numbered, sealed, opaque envelopes; or sequentially numbered, identical containers. Studies were considered to have a “high risk” of bias if participants were assigned to treatment conditions using a random sequence known to staff in advance, envelopes or packaging without safeguards, or a non-random, predictable sequence.

For blinding of participants and personnel, studies were considered to have a “low risk” of bias if measures to blind all parties from treatment allocation were taken, and it was unlikely that the blinding could have been broken. Studies were considered to have a “high risk” of bias if there was no blinding of allocation, incomplete or broken blinding, and the outcome of ANC/PNC adherence was likely to be influenced. For blinding of outcome assessment, criteria of “low risk” and “high risk” of bias followed the criteria established for blinding of participants and personnel, but were assessed in relation to the measurement of ANC/PNC adherence.

For incomplete outcome data, studies were considered to have a “low risk” of bias if there were no missing data, reasons for missing data were not related to the outcome of ANC/PNC adherence, missing data were balanced across groups, or if the proportion of missing data was not large enough to have a clinically relevant effect. Studies were considered to have a “high risk” of bias if reasons for missing data were related to ANC/PNC adherence, and there was an imbalance in numbers or reasons; the proportion of missing data was large enough to have a clinically relevant effect; an ‘as-treated’ analysis was used with substantial departure from original treatment allocation; or imputation was used inappropriately.

For selective reporting, studies were considered to have a “low risk” of bias if a study protocol was established and available prior to conducting the study, and all pre-specified outcomes of interest were reported in the pre-specified way; in cases where the protocol was not available, studies were determined to be “low risk” if it was clear that all pre-specified and expected outcomes of interest were reported. Studies were considered to have a “high risk” of bias if outcomes were not reported as pre-specified or expected or if outcomes were reported incompletely.

For other sources of bias, studies were considered to have a “low risk” of bias if the study appeared to be free of other sources of risk. Studies were considered to have a “high risk” of bias if there were issues specific to the study design (e.g. recruitment bias in cluster-randomized trials), baseline imbalance, or any additional sources of bias.

Across domains, studies were considered to have “unclear risk” of bias if there were insufficient details included to make a determination. Any disagreements regarding the qualitative ratings of bias within each category were reconciled by the third independent reviewer (JH). Judgments made within each domain were then synthesized at the study level with studies considered to have: “low risk” if there was a low risk of bias for all key domains; “unclear risk” if there was low or unclear risk of bias for all key domains; and “high risk” if there was a high risk of bias for one or more key domains.

## Results

**Fig. 1:**
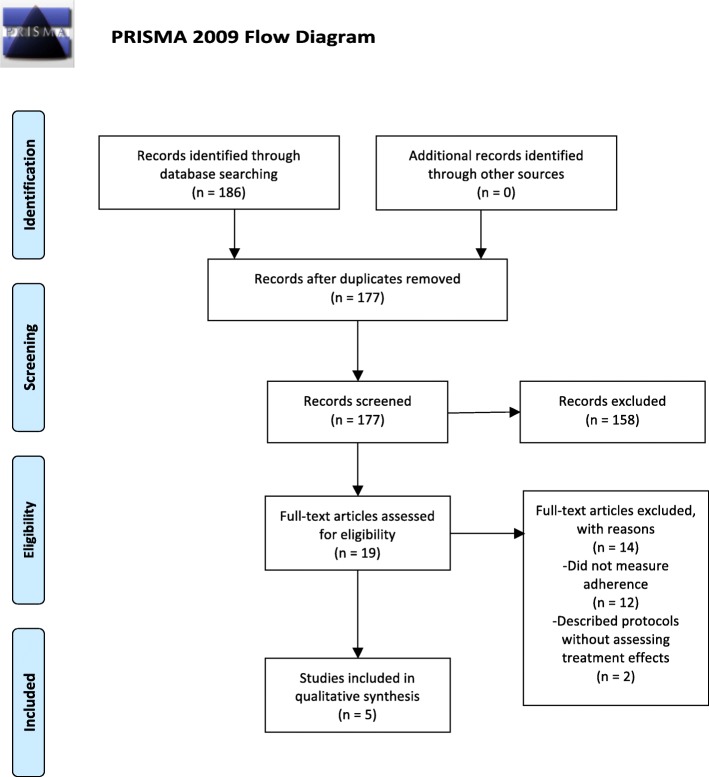
PRISMA Flow Diagram. Describes the number of articles identified, included and excluded, and the reasons for exclusions

Following the PRISMA guidelines [10], Fig. 1 illustrates the process for selecting studies that were included in this systematic review. The initial search identified 186 abstracts. After screening these abstracts, 19 studies were accepted for full-text review based on our PICO search strategy. Subsequently, 5 of these studies were still eligible after full text review and data extraction. The other 14 studies were rejected because they did not report treatment effects on ANC or PNC adherence as an outcome of interest.

Table 1 provides a high-level overview of findings from this systematic review. Overall, three ANC/PNC home visit interventions had no effect on ANC attendance, with two out of the three studies judged to have an “unclear risk” of bias and the third considered to have a “high risk” of bias. On the contrary, two behavioral interventions increased ANC uptake and PNC utilization, respectively, with one study assessing ANC judged to have an “unclear risk” of bias and the other assessing PNC considered to have a “high risk” of bias.

**Table 1:** Summary of Overall Findings

**Author, Date, Country**	**Intervention**	**Effectiveness**	**Quality of Evidence**
Ayiasi, 2016, Uganda	2 ANC home visits & 1 PNC home visit 3 days after delivery	No effect on ANC attendance	Unclear risk
Cherniak, 2017, Uganda	Advertising portable ultrasound by radio messaging	Increased ANC uptake	Unclear risk
Kirkwood, 2013, Ghana	2 ANC home visits & 3 PNC home visits after birth on days 1, 3, & 7	No effect on ANC attendance	Unclear risk
Magoma, 2013, Tanzania	Introduction & promotion of birth plans during ANC	Increased PNC utilization	High risk
Waiswa, 2015, Uganda	2 ANC home visits & 3 PNC home visits after birth on days 1, 3, &7	No effect on ANC attendance	High risk

Provides an overview of interventions, effectiveness, and quality of evidence

Table 2 summarizes the study design, intervention characteristics, and findings for each of the five studies included in this review [17–21]. Because of the small sample size of publications included and the differences across studies in interventions and adherence measures, there was little commonality to quantify differences between groups or calculate effect sizes that would allow comparisons of findings across studies. Thus, the results of the studies presented in Table 1 and discussed below, outline whether there were statistically significant differences in adherence to ANC/PNC between the treatment arms being compared within each individual study.

**Table 2:** Overview of Included Studies

**Author, Date, Country**	**StudyPopulation**	**Study Design**	**Intervention Strategy**	**Primary & Secondary Outcomes, Measurement of ANC/PNC Adherence**	**Results**	**Interpretation**
Ayiasi, 2016, Uganda	Pregnant women (Masindi & Kiryandongo districts)	Cluster RCT	Treatment (n=627): In addition to routine educational messages in ANC clinic, received village health teams (VHTs) making home visits to provide educational messages for maternal/newborn care & each VHT had mobile phone handset capable of making unlimited phone consultation with health workers; VHTs made 2 ANC visits (4 weeks apart) & 1 PNC home visit 3 days after delivery. Control (n=758): Received group education routinely offered in health centers, but did not receive VHT home visits or mobile phones. Follow-up: Data were collected 4-6 months after delivery.	Primary: Health facility delivery. Secondary: ANC attendance, birth preparation, cord care, thermal care, initiation of exclusive breastfeeding, & care-seeking for newborn illness. ANC attendance was measured through self-report where attending three or more ANC visits was categorized as adequate & the rest were grouped as inadequate.	85% of the intervention group made 3+ antenatal visits, compared to 71% of the control, adjusted odds ratio 1.82 (95% CI 0.65-5.09, p=0.26)	Home visit intervention had no effect on recommended ANC attendance.
Cherniak, 2017, Uganda	Pregnant women (Kabale district)	Cluster RCT	Treatment (n=100): Word of mouth ANC advertisement carried out by local community leaders who announced free ANC at community gatherings, plus advertisement for availability of portable ultrasound (pOBU), further divided into word of mouth of pOBU & ANC (n=16), radio advertisement of only ANC & word of mouth of ANC & pOBU (n=7), or word of mouth plus radio of both ANC & pOBU (n=75). Control (n=59): Word of mouth advertisment of ANC only with no mention of pOBU. Follow-up: Data were collected upon arrival to clinic and leaving clinic.	Primary: ANC attendance/uptake. Secondary: Attendance by women who previously used a traditional healer/birth attendant (TBA), had not yet attended ANC, attendance between three interventions, & # of women stating they came for pOBU. ANC uptake rate calculated using # of women attending ANC as the numerator & number of women attending first ANC in 2013-2014 through government-run clinics as the denominator.	Rate of ANC attendance was 65.1% per 1000 pregnant women where pOBU advertised by radio & word of mouth vs. 11.1% in control communities, rate ratio 5.86 (95% CI 2.6-13.0, p<0.0001)	Advertising pOBU by radio messaging significantly increased ANC uptake as compared to word of mouth advertisement of ANC only.
Kirkwood, 2013, Ghana	Pregnant women (Brong Ahafo region)	Cluster RCT	Treatment (n=9174): Integrated intervention training community based surveillance volunteers (CBSVs) to identify pregnant women in their community, undertake 2 home visits during pregnancy & 3 visits after birth on days 1, 3, & 7 in addition to standard care provided. CBSVs also were responsible to weigh the newborn & check them for danger signs after birth. Control (n=9435): Standard care available, including antenatal clinics, access to free facility delivery, post-partum checkups, infant welfare clinics, & routine CBSV activities for outreach. Follow-up: Data were collected via surveillance home visits that occurred every 4-8 weeks throughout the study period.	Primary: All-cause neo-natal mortality rate (NMR) in the 1st 28 days of life & % of mothers practicing Newhints recommended behaviors (including # of ANC visits). Secondary: Age-specific & cause-specific NMRs. Attendance to 4 or more ANC visits was measured via self-report following birth.	76% treatment & 73.7% control attended 4 or more ANC visits (2.3% incr.), relative risk 1.02 (95% CI 0.96-1.09, p=0.52)	Home visit intervention had no effect on recommended ANC attendance.
Magoma, 2013, Tanzania	Pregnant women (Ngorongoro district, Arusha region)	Cluster RCT	Treatment (n=404): Introduction & promotion of birth plans by care providers during ANC to prepare women & families for birth. Discussions on place of delivery, importance of skilled delivery care, transport arrangements, funding, possible blood donors, birth companions, & home support. Control (n=501): Standard care without birth plan. Follow-up: Data were collected within 1 month of delivery.	Primary: Delivery in health unit. Secondary: PNC attendance, satisfaction of women & providers with care received & provided. PNC attendance within 1 month of delivery was determined from self-reports & cross-checked using health records.	PNC utilization within 1 month: 62.1% in treatment & 32.1% in control, adjusted absolute difference 31.3% (95% CI 15.4-47.2, p=0.0009); days to initial PNC (mean +- SD) treat 6.6 +-1.7 vs. control 20.9+-4.4, p=0.0001	Introduction & promotion of birth plans during ANC care increased PNC utilization in the first month after delivery.
Waiswa, 2015, Uganda	Pregnant women (Iganga & Mayuge districts)	Cluster RCT	Treatment (n=894): 5 home visits by community health workers, 2 during pregnancy & 3 in the 1st week after birth (day 1, 3, & 7) to offer preventative & promotive care/counseling with extra visits for sick & small newborns to assess & refer plus improved facilities Control (n=893): Standard care overseen by district health team in addition to improved facilities. Follow-up: Endline data were collected amongst women who had a live birth within 12 months of the baseline survey.	Primary: Coverage of ANC, birth preparedness, skilled attendance at delivery, PNC, breastfeeding, thermal care, & hygiene. Secondary: None reported. Data on attendance to one or more ANC visits and to four or more ANC visits were collected via self-report.	99.2% intervention & 98.9% control attended at least one ANC visit, p=0.44); 47% intervention & 43.6% control attended 4 or more ANC visits, p=0.165	Home visit intervention had no effect on recommended ANC attendance.

All of the studies were cluster randomized controlled trials of interventions to improve maternal and infant health that took place within the past six years. Three were conducted in Uganda [17, 18, 21], one in Ghana [19], and one in Tanzania [20]. Although ANC or PNC adherence was measured in each study, the primary outcome of interest varied across studies, with two focusing on delivery in a health facility [17, 20], one on coverage of key essential newborn care behaviors, such as breastfeeding, thermal care, and cord care [21], one on neonatal mortality and newborn care behaviors [19], and one on ANC attendance explicitly [18].

Three studies implemented a home visit intervention in which trained community health workers visited the homes of identified pregnant women and provided educational counseling on preventive and promotive care during pregnancy [17, 19, 21]. Two of the three included two prenatal and three postnatal home visits [19, 21], while the remaining study included two prenatal home visits and one postnatal home visit [17]. A variety of topics related to preventive and promotive care were covered in the visits. Prenatal visits focused on danger signs in pregnancy, birth preparation, and clean delivery practices promoting the health of the newborn, including hygienic cord care, proper wrapping, early/exclusive breastfeeding, and delayed bathing. Postnatal visits focused on screening for and counseling on maternal and newborn danger signs, as well as encouragement and reinforcement of breastfeeding, skin-to-skin contact, newborn immunization, and prompt care-seeking. In each study, there was no statistically significant difference in ANC attendance between the treatment group that received the home visit intervention and the standard care control group (see Table 2). Importantly, ANC adherence to the WHO recommendations of four ANC visits during pregnancy remained fairly low across treatment arms in the two studies that measured this particular outcome, with rates of 74% adherence amongst the control and 76% amongst the treatment group in the Kirkwood et al. study[19], and rates of 44% adherence amongst the control and 47% amongst the treatment group in the Waiswa et al. study [21].

The remaining two studies implemented behavioral interventions that used planning and incentive schemas, respectively, to promote ANC/PNC uptake among pregnant women. In the Magoma et al. study, ANC healthcare providers helped pregnant women in the treatment group to develop a birth plan prior to delivery [20]. Developing a birth plan involved discussions on place of delivery, importance of skilled delivery care, transport arrangements, funding, possible blood donors should an emergency occur, birth companions, and home support. As a result, PNC utilization in the first month after delivery was higher amongst the intervention group compared to the standard care control group (62% vs. 32%, respectively; 95% CI 15.4 - 47.2, p = 0.0009). Further, women in the treatment group sought PNC approximately three times sooner than those in the control group (see Table 2).

In the final included study [18], the treatment group was exposed to advertisement for ANC, and informed about the availability of a portable ultrasound (pOBU), in three separate conditions: 1) word of mouth advertisement of ANC and pOBU; 2) word of mouth advertisement of ANC and pOBU plus radio advertisement of only ANC; and 3) word of mouth advertisement of ANC and pOBU plus radio advertisement of both ANC and pOBU. ANC uptake was significantly higher among those subjected to word of mouth plus radio advertisement of ANC and pOBU compared to the control group that received word of mouth advertisment of ANC only with no mention of pOBU (65% vs. 11%, respectively, rate ratio 5.86, 95% CI 2.6 - 13.0, p < 0.0001; see Table 2). There were no differences in rate ratio attending ANC among the three variants of the intervention arm, and when comparing each of the first two intervention variants to the control group.

Thus, overall, two of the five studies [18, 20] included in this review demonstrated the effectiveness of behavioral interventions to increase ANC/PNC attendance. The remaining three studies, which implemented a home visit intervention, did not seem to have an impact on adherence to recommended ANC regimens [17, 19, 21].

Table 3 summarizes the results from the Cochrane risk of bias assessment (see Additional file 3 for comprehensive evidence supporting each judgment). Risk of bias was low in most categories across studies. All five studies received “low risk” ratings for selection bias by documenting the use of random sequence generation and adequate treatment allocation concealment. All five studies were also judged to be “low risk” for attrition bias based on the extent of missing data from each group, the reasons provided, and the type of analysis conducted. When evaluating detection bias via blinding of outcome assessors from knowledge of which intervention a participant received, all studies received “unclear risk” ratings due to lack of information provided. The greatest variation across studies was in the performance bias domain, assessing blinding of participants and personnel to treatment allocation, with one study rated as “low risk” because the researchers intentionally masked the presence of pOBU when initial consent was obtained in the control arm [18]; three as “unclear risk” because they do not mention or use a procedure to blind participants and study personnel to treatment allocation [17, 19, 21]; and one as “high risk” because it did not allow blinding of birth plan providers or pregnant women who participated in the study to the treatment allocation [20]. To examine reporting bias, each protocol was checked for discrepancies between outcomes of interest study authors said they would measure and those they report on; three of the five studies [17–19] received “low risk” ratings in selective reporting, however, the Waiswa et al. study [21] was rated as “high risk” in this category due to the presence of pre-specified intermediate outcomes that appear to be excluded in the published paper. Finally, sources of other bias were “low risk” in four out of five studies [18–21], with one study judged to have “unclear risk” due to baseline imbalance between treatment arms [17].

**Table 3:** Risk of Bias Within Studies

**Author, Date, Country**	**Random Sequence Generation (Selection Bias)**	**Treatment Allocation Concealment (Selection Bias)**	**Blinding of Participants and Personnel (Performance Bias)**	**Blinding of Outcome Assessment (Detection Bias)**	**Incomplete Outcome Data (Attrition Bias)**	**Selective Reporting (Reporting Bias)**	**Other Sources of Bias**
Ayiasi, 2016, Uganda	Low risk	Low risk	Unclear risk	Unclear risk	Low risk	Low risk	Unclear risk
Cherniak, 2017, Uganda	Low risk	Low risk	Low risk	Unclear risk	Low risk	Low risk	Low risk
Kirkwood, 2013, Ghana	Low risk	Low risk	Unclear risk	Unclear risk	Low risk	Low risk	Low risk
Magoma, 2013, Tanzania	Low risk	Low risk	High risk	Unclear risk	Low risk	Low risk	Low risk
Waiswa, 2015, Uganda	Low risk	Low risk	Unclear risk	Unclear risk	Low risk	High risk	Low risk

Summarizes results from the Cochrane risk of bias assessment

Synthesizing the overall level of evidence across domains, three of the five studies [17–19] included in this review were considered to have an “unclear risk” of bias, while the remaining two studies [20, 21] had a “high risk” of bias.

## Discussion

Given high rates of infant and maternal mortality in sub-Saharan Africa, the purpose of this systematic review was to examine the availability of effective behavioral interventions to increase adherence to ANC and PNC regimens among pregnant women, which have the potential to improve maternal and newborn health outcomes. Of the five studies reviewed, two demonstrated effectiveness in increasing ANC or PNC uptake [18, 20]; both studies implemented behavioral interventions using incentives/reminders and planning, respectively. Three community health worker home visit interventions had no effect on ANC adherence [17, 19, 21]. Taking the potential risk of systematic bias into account, the paucity of studies identified in this review reveals a gap in evidence-based interventions to increase ANC/PNC adherence in sub-Saharan Africa. Nonetheless, the positive impact of behavioral interventions to increase ANC/PNC adherence in this review complements previous work documenting the effects of reminder- and planning-based mobile health interventions on ANC/PNC adherence and maternal and neonatal health outcomes [22–25].

We speculate that Cherniak et al. [18] and Magoma et al. [20] found effects of their interventions because the interventions targeted mothers’ high motivation to adhere to ANC/PNC regimens, as well as cognitive processes vital to adherence, such as memory, planning, and task monitoring [26]. Consistent with research demonstrating the positive impact of mass media on the utilization of ANC [27–31], Cherniak et al. showed that using the radio to advertise the availability of a pOBU during ANC significantly increases ANC uptake [18]. Although the effect can be explained as a simple incentive effect because of the free provision of pOBU, it is also possible that the pOBU acted as a salient reminder to attend ANC in this low-income context, where fewer tasks have built-in reminders. Indeed, Mullainathan and Shafir [32] have documented the effects of “scarcity”, a cognitive form of stress induced by contexts of limited resources, that produces characteristic flaws in executive function. In line with this view, text message reminders have been linked to greater ANC and PNC adherence in developing contexts [24]. Further, Magoma et al. [20] showed that the introduction and promotion of birth plans by care providers during ANC significantly increases PNC utilization, suggesting that the engagement of executive control through the act of planning ahead for the delivery of the baby can impact adherence to PNC regimens. This finding is consistent with research on implementation intentions, demonstrating that the realization of a goal is more likely to be achieved by forming a plan that describes the when, where, and how of goal striving in advance [33, 34].

The three studies that did not find effects on ANC adherence implemented a home visit intervention, where community health workers educated pregnant women on danger signs in pregnancy, birth preparation, and clean, newborn health-promoting delivery practices, including hygienic cord care, proper wrapping, early/exclusive breastfeeding, and delayed bathing; and screened for and counseled on maternal and newborn danger signs, breastfeeding, skin-to-skin contact, newborn immunization, and prompt care-seeking. Because women received two ANC visits at their homes, the null results might reflect that mothers considered attending external ANC redundant (despite the fact that the WHO recommends that pregnant women attend at least eight ANC visits prior to delivery). Alternatively, these studies may have underestimated the treatment effects given that the measures of adherence relied on self-reports, a method that is known to overestimate adherence [35–37] and thus could potentially lead to a ceiling effect in estimating differences between groups. Further, while Ayiasi et al. [17] and Kirkwood et al. [19] found that approximately 75% of the sample adhered to at least three ANC visits, suggesting that ANC adherence is relatively high overall, Waiswa et al. [21] reported that less than half of the sample adhered to the previous WHO recommendations, thus highlighting the need for further studies on interventions to improve ANC adherence directly.

Like other qualitative reviews of intervention studies, this systematic review has its limitations. The goal of this review was to identify all studies that met the eligibility criteria, but it is possible that we have missed relevant articles indexed in other databases than those chosen for review. Future reviews on this topic could be strengthened by scanning additional databases, such as Embase, MEDLINE, and Cochrane Central, as well as the reference lists of included publications. Further, the gold standard for reviewing intervention effectiveness is with a meta-analysis that calculates a pooled effect size from RCTs. In this review, the interventions and adherence measures were too heterogeneous to combine in a meta-analysis. Instead, we present (1) simple summary data for each intervention group and (2) effect estimates and confidence intervals for each study, following PRISMA guidelines [10]. Thus, although each individual trial utilizing a home visit intervention was powered to detect an effect on ANC adherence with no effect found, it is impossible to draw pooled conclusions from the current review.

Further, while the scope of the current review focused on the effectiveness of interventions to improve ANC/PNC attendance specifically, there is increasing discussion in the literature emphasizing the difference between “contacts” (i.e. number of visits) and “content” of ANC/PNC, as the quality of care is important to consider alongside the quantity of care. A recent meta-analysis demonstrated that neonatal mortality was significantly lower among children of women who received high-quality ANC by skilled personnel [38]. Efforts to increase utilization of services alone can often be accompanied by poor or declining quality of service, which may in turn reduce future utilization of services [39]. Indeed, Duysburgh et al. [40] identified critical gaps in counseling and health education practices, laboratory investigations, examination and monitoring of mother and newborn during childbirth, and necessary birth equipment, reducing the quality of ANC and childbirth care in Burkina Faso, Ghana, and Tanzania. Similar inadequacies in care have been demonstrated in Zambia [41], Kenya [42], and across sub-Saharan Africa [43]. Thus, boosting pregnant women’s attendance to ANC/PNC services is likely to be necessary, but not sufficient to improve infant and maternal health outcomes absent significant focus on quality of care.

## Conclusions

The present study raises several questions for future research. There appear to be very few interventions that aim to directly improve ANC/PNC adherence in sub-Saharan Africa, despite high rates of infant and maternal mortality that persist in this region. Access to free ANC/PNC in developing contexts is only effective in reducing infant and maternal mortality when mothers attend these clinics, yet ANC attendance was the primary outcome of interest in only two out of five included studies. Interventions drawing upon the executive function literature and the promising results of the behavioral interventions reviewed here are urgently needed, alongside assessments of quality of care, to address these gaps.

## Supplementary information

**Additional file 1** PRISMA checklist.

**Additional file 2** PROSPERO systematic review protocol.

**Additional file 3** Cochrane risk of bias determination.

## Data Availability

All data used and analyzed during the current study are available from the corresponding author on reasonable request.

## Abbreviations

ANC: antenatal care
PNC: postnatal care
WHO: World Health Organization
RCT: randomized controlled trial
PICO: Population Intervention Comparison Outcome (search strategy)
PRISMA: Preferred Reporting Items for Systematic reviews and Meta-Analyses
pOBU: portable ultrasound
