# Supplementary material for: Interventions to improve adherence to antenatal and postnatal care regimens among pregnant women in sub-Saharan Africa: a systematic review
Source: BMC Pregnancy Childbirth. 2020 May 24;20:316. doi: 10.1186/s12884-020-02992-y (PMC7245828; doi:10.1186/s12884-020-02992-y)
Supplement: Supplementary file 2 — Additional file 2 PROSPERO systematic review protocol. [file 12884_2020_2992_MOESM2_ESM.pdf]

Interventions to improve adherence to antenatal and postnatal care regimens among pregnant women in sub-Saharan Africa: a systematic review

Kristina Esopo, Lilly Derby, Johannes Haushofer

Citation

Kristina Esopo, Lilly Derby, Johannes Haushofer. Interventions to improve adherence to antenatal and postnatal care regimens among pregnant women in sub-Saharan Africa: a systematic review. PROSPERO 2018 CRD42018088152 Available from:
https://www.crd.york.ac.uk/prospero/display_record.php?ID=CRD42018088152

Review question

Which existing interventions are effective at improving adherence to the recommended number of antenatal/postnatal doctor visits in sub-Saharan Africa?

Searches

PubMed: all studies in Sub-Saharan Africa published through the date the searches are run, restricted to RCTs only; PsycINFO: all studies in Sub-Saharan Africa published through the date the searches are run, restricted to English, peer-reviewed, and RCTs only

PubMed Search Strategy: 713 results, 97 results when restricting to RCTs only
(“intervention“[Title/Abstract] OR “program”[Title/Abstract] OR “training”[Title/Abstract]) AND (“Africa”[Title/Abstract] OR “Angola”[Title/Abstract] OR “Benin”[Title/Abstract] OR “Botswana”[Title/Abstract] OR “Burkina Faso”[Title/Abstract] OR “Burundi”[Title/Abstract] OR “Cabo Verde”[Title/Abstract] OR “Cameroon”[Title/Abstract] OR “Central African Republic”[Title/Abstract] OR “Chad”[Title/Abstract] OR “Comoros”[Title/Abstract] OR “Congo”[Title/Abstract] OR “Côte d'Ivoire”[Title/Abstract] OR “Cote d’Ivoire”[Title/Abstract] OR “Eritrea”[Title/Abstract] OR “Ethiopia”[Title/Abstract] OR “Gabon”[Title/Abstract] OR “Gambia”[Title/Abstract] OR “Ghana”[Title/Abstract] OR “Guinea”[Title/Abstract] OR “Kenya”[Title/Abstract] OR “Lesotho”[Title/Abstract] OR “Liberia”[Title/Abstract] OR “Madagascar”[Title/Abstract] OR “Malawi”[Title/Abstract] OR “Mali”[Title/Abstract] OR “Mauritania”[Title/Abstract] OR “Mauritius”[Title/Abstract] OR “Mozambique”[Title/Abstract] OR “Namibia”[Title/Abstract] OR “Niger”[Title/Abstract] OR “Nigeria”[Title/Abstract] OR “Rwanda”[Title/Abstract] OR “São Tomé and Principe”[Title/Abstract] OR “Sao Tome and Principe”[Title/Abstract] OR “Senegal”[Title/Abstract] OR “Seychelles”[Title/Abstract] OR “Sierra Leone”[Title/Abstract] OR “Somalia”[Title/Abstract] OR “South Africa”[Title/Abstract] OR “South Sudan”[Title/Abstract] OR “Sudan”[Title/Abstract] “Swaziland”[Title/Abstract] OR “Tanzania”[Title/Abstract] OR “Togo”[Title/Abstract] OR “Uganda”[Title/Abstract] OR “Zambia”[Title/Abstract] OR “Zimbabwe”[Title/Abstract]) AND (“prenatal”[Title/Abstract] OR “postnatal”[Title/Abstract] OR “antenatal”[Title/Abstract] OR “pregnant”[Title/Abstract] OR “adherence”[Title/Abstract])

PsycINFO Search Strategy: 677 results (English and peer-reviewed only), 89 results when adding RCT descriptives in search terms

(AB “intervention“ OR “program” OR “training”) AND (AB “Africa” OR “Angola” OR “Benin” OR “Botswana” OR “Burkina Faso” OR “Burundi” OR “Cabo Verde” OR “Cameroon” OR “Central African Republic” OR “Chad” OR “Comoros” OR “Congo” OR “Côte d'Ivoire” OR “Cote d’Ivoire” OR “Eritrea” OR “Ethiopia” OR “Gabon” OR “Gambia” OR “Ghana” OR “Guinea” OR “Kenya” OR “Lesotho” OR “Liberia” OR “Madagascar” OR “Malawi” OR “Mali” OR “Mauritania” OR “Mauritius” OR “Mozambique” OR “Namibia” OR “Niger” OR “Nigeria” OR “Rwanda” OR “São Tomé and Principe” OR “Sao Tome and Principe” OR “Senegal” OR “Seychelles” OR “Sierra Leone” OR “Somalia” OR “South Africa” OR “South Sudan” OR “Sudan” “Swaziland” OR “Tanzania” OR “Togo” OR “Uganda” OR “Zambia” OR “Zimbabwe”) AND (AB “prenatal” OR “postnatal” OR “antenatal” OR “pregnant” OR “adherence”) AND (AB “randomized controlled trial” OR “randomized trial” OR “RCT” OR “randomized”)

Types of study to be included
We will include only randomized controlled trials.

Condition or domain being studied
Antenatal and postnatal adherence among pregnant women; outcomes must include adherence to doctor visits; maternal and infant health outcomes will be assessed if included but not required.

Participants/population
Inclusion: Pregnant women in sub-Saharan Africa.

Intervention(s), exposure(s)
Interventions must aim to improve pregnant women's adherence to the recommended number of antenatal and postnatal visits, with potential assessment of intervention impact on maternal and infant health/mortality.

Comparator(s)/control
Control group without intervention.

Context
Only relevant research conducted in sub-Saharan Africa will be included.

Main outcome(s)
Adherence to the recommended number of doctor visits, measured as the difference between treatment and control.

Additional outcome(s)
Maternal and infant mortality, measured by: mortality rates, apgar scores for the baby at 1 and 5 minutes after birth, and other health outcomes across treatment and control groups

Data extraction (selection and coding)
Titles and abstract of studies retrieved using the search strategy and those from additional sources will be screened independently by two review authors to identify studies that potentially meet the inclusion criteria outline above. Using "abstrackr", study selection will be partially blinded such that the researchers are unaware of journal details. The full text of these potentially eligible studies will be retrieved and independently assessed for eligibility by two review team members. Any disagreement between them over the eligibility of particular studies will be resolved through discussion with a third independent reviewer.

A standardised, pre-piloted form will be used to extract data from the included studies for assessment of study quality and evidence synthesis. Extracted information will include: study setting; study population and participant demographics and baseline characteristics; details of the intervention and control conditions, including number of participants; study methodology; recruitment and study completion rates; outcomes and times of measurement; treatment effects; information for assessment of the risk of bias. Two review authors will extract data independently, discrepancies will be identified and resolved through discussion (with a third author where necessary). Missing data will be requested from study authors.

Risk of bias (quality) assessment
Two review authors will independently assess the risk of bias in included studies, using the Cochrane risk of bias tool. The following characteristics will be considered:

1. Randomization sequence generation: was the allocation sequence adequately generated?

2. Treatment allocation concealment: was the allocated treatment adequately concealed from study participants and clinicians and other healthcare or research staff at the enrollment stage?

3. Blinding: were the personnel assessing outcomes and analysing data sufficiently blinded to the intervention allocation throughout the trial?

4. Completeness of outcome data: were participant exclusions, attrition and incomplete outcome data adequately addressed in the published report?

5. Selective outcome reporting: is there evidence of selective outcome reporting and might this have affected the study results?

6. Other sources of bias: was the trial apparently free of any other problems that could produce a high risk of bias?

Disagreements between the review authors over the risk of bias in particular studies will be resolved by discussion, with involvement of a third review author where necessary.

Strategy for data synthesis

We will provide a narrative synthesis of the findings from the included studies, structured around the type of intervention, target population characteristics, type of outcome and intervention content. We will provide summaries of intervention effects for each study by calculating risk ratios (for dichotomous outcomes) or standardised mean differences (for continuous outcomes).

We anticipate that there will be limited scope for meta-analysis because of the range of different outcomes measured across the small number of existing trials. However, where studies have used the same type of intervention and comparator, with the same outcome measure, we will pool the results using a random-effects meta-analysis, with standardised mean differences for continuous outcomes and risk ratios for binary outcomes, and calculate 95% confidence intervals and two sided P values for each outcome. In studies where the effects of clustering have not been taken into account, we will adjust the standard deviations for the design effect. Heterogeneity between the studies in effect measures will be assessed using both the ?2 test and the I^2 statistic. We will consider an I^2 value greater than 50% indicative of substantial heterogeneity. We will conduct sensitivity analyses based on study quality. We will use stratified meta-analyses to explore heterogeneity in effect estimates according to: study quality; study populations; the logistics of intervention provision; and intervention content. We will also assess evidence of publication bias.

Analysis of subgroups or subsets

This is a qualitative synthesis and while subgroup analyses may be undertaken it is not possible to specify the groups in advance.

Contact details for further information

Kristina Esopo
kesopo@princeton.edu

Organisational affiliation of the review

Princeton University

Review team members and their organisational affiliations

Miss Kristina Esopo. Department of Psychology, Princeton University
Miss Lilly Derby. Center for Behavioral Cardiovascular Health, Columbia University Medical Center
Assistant/Associate Professor Johannes Haushofer. Department of Psychology and Woodrow Wilson School of Public and International Affairs, Princeton University

Type and method of review

Intervention, Qualitative synthesis, Systematic review

Anticipated or actual start date

25 January 2018

Anticipated completion date

09 April 2018

Funding sources/sponsors
Funded NIH proposal: 5UH2NR01637803

Conflicts of interest

Language
English

Country
United States of America

Stage of review
Review Ongoing

Subject index terms status
Subject indexing assigned by CRD

Subject index terms
Africa South of the Sahara; Female; Humans; Postnatal Care; Pregnancy; Pregnant Women

Date of registration in PROSPERO
07 February 2018

Date of publication of this version
20 March 2018

Details of any existing review of the same topic by the same authors

Stage of review at time of this submission

Stage	Started	Completed
Preliminary searches	Yes	No
Piloting of the study selection process	Yes	No
Formal screening of search results against eligibility criteria	No	No
Data extraction	No	No
Risk of bias (quality) assessment	No	No
Data analysis	No	No

Versions
07 February 2018
20 March 2018

PROSPERO
This information has been provided by the named contact for this review. CRD has accepted this information in good faith and registered the review in PROSPERO. The registrant confirms that the information supplied for this submission is accurate and complete. CRD bears no responsibility or liability for the content of this registration record, any associated files or external websites.
